# If Loneliness Is a Public Health Issue, Then What Is a Public Health Approach to Loneliness?

**DOI:** 10.1093/geroni/igaf122.195

**Published:** 2025-12-31

**Authors:** Roger O’Sullivan

**Affiliations:** The Institute of Public Health, Belfast/Dublin, Northern Ireland, United Kingdom

## Abstract

The U.S. Surgeon General and the World Health Organization (WHO) have highlighted the significant impact loneliness has on the health and well-being of the population, and loneliness is increasingly being recognized as a public health issue. However, there is less discussion on how to drive a public health approach to loneliness. This presentation outlines a public health framework to address loneliness, first setting out the context and methods of public health, as well as the steps of a public health approach. By adapting the Frieden Health Impact Pyramid and its five tiers to the issue of loneliness, the types of actions required at each tier are identified to address loneliness among older people. At the base of the pyramid, actions have the widest population focus, while those further up the pyramid have a greater individual focus. Tier 1 actions address structural factors. Tier 2 highlights interventions which aim to change the context. Tier 3 focuses on protective interventions. Tier 4 provides examples of tailored interventions for individuals, and Tier 5 outlines specialized interventions. In summary, to advance a public health approach to loneliness among older people, increasing focus should be placed on: 1) preventing loneliness; 2) protecting those groups most at risk; and 3) promoting a greater understanding of the causes and consequences of loneliness. The suggested public health actions at the structural, community, and individual levels aim to address the negative costs of loneliness, not only for lonely individuals but society as a whole.

